# Mitochondrial Magnesium is the cationic rheostat for MCU-mediated mitochondrial Ca^2+^ uptake

**DOI:** 10.21203/rs.3.rs-3088175/v1

**Published:** 2023-07-18

**Authors:** Thiruvelselvan Ponnusamy, Prema Velusamy, Amrendra Kumar, Daniel Morris, Xueqian Zhang, Gang Ning, Marianne Klinger, Jean E. Copper, Sudarsan Rajan, Joseph Y Cheung, Kalimuthusamy Natarajaseenivasan, Nelli Mnatsakanyan, Santhanam Shanmughapriya

**Affiliations:** 1 Heart and Vascular Institute, Department of Medicine, Department of Cellular and Molecular Physiology, Pennsylvania State University, College of Medicine, Hershey, PA 17033, USA; 2 Department of Cellular and Molecular Physiology, Pennsylvania State University, College of Medicine, Hershey, PA 17033, USA; 3 Cardiovascular Medicine, Department of Medicine, UMass Chan Medical School, Worcester, MA 01655, USA; 4 Microscopy Core Facility, Penn State Huck Institutes of the Life Sciences, University Park, PA 16802, USA; 5 Department of Pathology, Pennsylvania State University, College of Medicine, Hershey, PA 17033, USA; 6 Center for Translational Medicine, Lewis Katz School of Medicine, Temple University, Philadelphia, PA 19140, USA.; 7 Department of Renal Medicine, Brigham & Women’s Hospital, Harvard Medical School, Boston, MA 02115, USA; 8 Department of Neural Sciences, Lewis Katz School of Medicine, Temple University, Philadelphia, PA 19140, USA.

## Abstract

Calcium (Ca^2+^) uptake by mitochondria is essential in regulating bioenergetics, cell death, and cytosolic Ca^2+^ transients. Mitochondrial Calcium Uniporter (MCU) mediates the mitochondrial Ca^2+^ uptake. MCU is a heterooligomeric complex with a pore-forming component and accessory proteins required for channel activity. Though MCU regulation by MICUs is unequivocally established, there needs to be more knowledge of whether divalent cations regulate MCU. Here we set out to understand the mitochondrial matrix Mg^2+^-dependent regulation of MCU activity. We showed Mrs2 as the authentic mammalian mitochondrial Mg^2+^ channel using the planar lipid bilayer recordings. Using a liver-specific Mrs2 KO mouse model, we showed that decreased matrix [Mg^2+^] is associated with increased MCU activity and matrix Ca^2+^ overload. The disruption of Mg^2+^dependent MCU regulation significantly prompted mitochondrial permeability transition pore opening-mediated cell death during tissue IR injury. Our findings support a critical role for mMg^2+^ in regulating MCU activity and attenuating mCa^2+^ overload.

## INTRODUCTION

Calcium (Ca^2+^) is a unique cellular ion and a critical regulator of multiple cellular processes, including mitochondrial function and dysfunction^1^. The simultaneous interplay of the on-and-off mechanisms maintains the intracellular Ca^2+^ (iCa^2+^) transients^2–7^. Such a fine regulation is mediated by mitochondria that can decode and transduce iCa^2+^ signals into an energy output (ATP) to match energetic cellular demand^1^. The Ca^2+^ selective channel, the Mitochondrial Ca^2+^ Uniporter (MCU), precisely controls mitochondrial Ca^2+^ (mCa^2+^) uptake and is driven by the considerable electrical gradient (Δψm) across the inner mitochondrial membrane (IMM)^8–11^. MCU functions as a hetero-oligomeric complex after self-association and interaction with its regulators^12–19^. Under physiological conditions, MCU-mediated mCa^2+^ uptake regulates bioenergetics by increasing the supply of reducing equivalents to the electron transport chain and increasing F1-F0 ATP synthase activity^1, 3, 20–22^. Reciprocally, in ischemia and reperfusion (I/R), dysregulation of mCa^2+^ cycling is prominent and results in either necrosis or apoptosis through mCa^2+^ overload^23–28^. Thus MCU-mediated mCa^2+^ uptake must be tightly regulated to support both cellular energy demand and also prevent cell death.

Though many Ca^2+^ channels, including InP_3_R^29^, RyRs^30^, and CRAC^31, 32^, exhibit Ca^2+^-dependent feedback mechanisms, whether divalent cations regulate MCU activity is unclear. To understand in depth the regulation of MCU by divalent cations, we resolved the atomic structure of the conserved MCU matrix domain^33^. Our previous structural analysis of the amino-terminal domain of human MCU revealed a β-grasplike fold containing the MCU regulating acidic patch (MRAP) that binds Mg^2+^/Ca^2+^ with ~mM affinity. The binding of Mg^2+^/Ca^2+^ to MRAP destabilizes MCU, shifts the self-association equilibrium to monomer, and attenuates mCa^2+^ uptake^33^. A seminal work from Foskett’s group showed the Ca^2+^ that permeates through the channel pore to bind the MRAP region and close the channel despite Ca^2+^ binding to MICU1/2^34^. Also, mutating the two key aspartic acid residues (D131 and D147) to alanine abolished matrix [Ca^2+^] dependent channel inhibition, validating divalent cation binding site encompassing D131 and D147 could account for Ca^2+^ (and Mg^2+^) dependent MCU inhibition^34^. Though matrix Ca^2+^ was shown to inhibit MCU channel activity, the weak binding affinity for divalent cation for MRAP is well suited to the high Mg^2+^ levels of the mitochondrial matrix (0.5–1.5 mM). Because proteins structurally sensitive to cations have evolved affinities close to the concentration range in the compartment where they function, we anticipate mitochondrial matrix [Mg^2+^] to regulate MCU channel activity tightly in addition to Ca^2+^.

Here we set out to understand the matrix [Mg^2+^] dependent regulation of MCU-channel activity. Using biochemical, lipid bilayer, and patch-clamp recordings, we show Mrs2 as the authentic mammalian mitochondrial Mg^2+^ channel. Using our newly developed liver-specific mouse model, we illustrate the loss of Mrs2 to drastically alter the matrix [Mg^2+^]. The decreased matrix [Mg^2+^] was associated with increased MCU activity and matrix Ca^2+^ overload. The disruption of Mg^2+^-dependent MCU activity also significantly prompted mitochondrial permeability transition pore opening (mPTP). We employed a hanging-weight system for liver ischemia/reperfusion (IR) to assess the loss of Mg^2+^-dependent MCU regulation and mPTP-mediated cell death during tissue IR injury. Our findings support a critical role for mMg^2+^ in regulating MCU activity and attenuating mCa^2+^ overload.

## RESULTS

**Figure 1.**
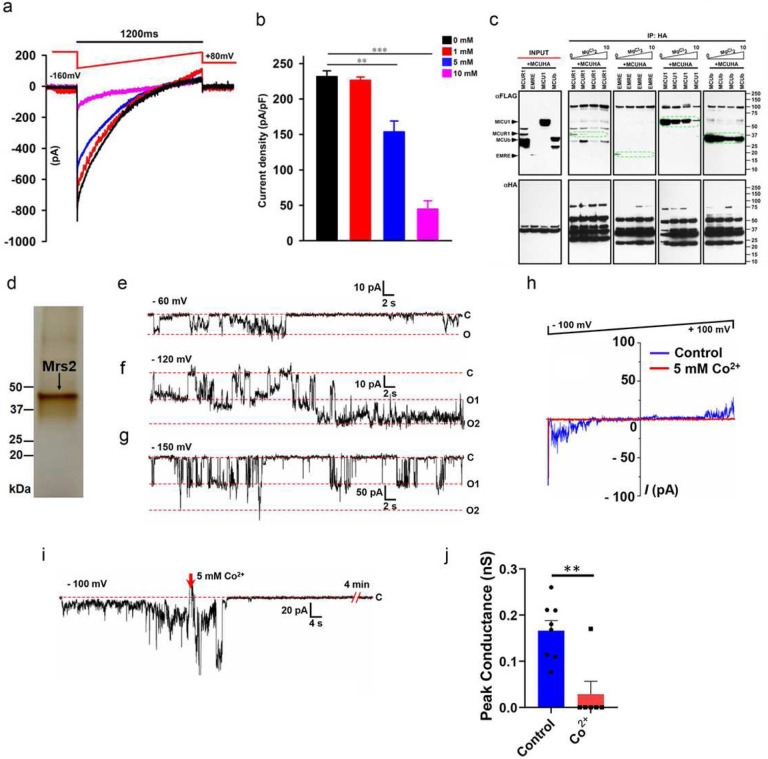
Matrix [Mg^2+^] regulates MCU activity, and mammalian Mrs2 is the authentic mitochondrial Mg^2+^ channel. (**a and b**) Measurement of *I*_*MCU*_ current in mitoplasts prepared from HeLa cells. *I*_*MCU*_ was measured with varying [Mg^2+^] (0, 1, 5, and 10 mM) in the patch-clamp pipette solution that filled the mitochondrial matrix. Traces are a single representative recording of *I*_*MCU*_. (**b**) *I*_*MCU*_ densities (pA/pF). Bar represents Mean ± SEM; **P < 0.01; ***P < 0.001; n = 4–7. (**c**) HEK293 cells were co-transfected with HAtagged MCU and Flag-tagged MICU1, MCUR1, MCUb, and EMRE. Following immunoprecipitation with HA antibody in the presence of varying concentrations of MgCl_2_ (0, 1, 5, and 10 mM), total cell lysates and immunoprecipitated materials were subjected to Western blot analysis. Cell lysates were probed with anti-Flag (top left panel) or anti-HA antibodies (bottom left panel) to serve as inputs. Immunoprecipitated samples were probed with anti-Flag (top right panels 1 to 4) and anti-HA antibodies (bottom right panels 1 to 4) (n=3). (**d**) Silver-stained gel of purified Mrs2 from HEK 293 cells. The single band at ~40 kDa position represents purified Mrs2. SDS-PAGE resolved the sample. (**e-g**) Representative channel recordings of purified Mrs2 in a lipid bilayer at −60, −120, and −150 mV are displayed in panels (**e**), (**f**), and (**g**), respectively. (**h**) Representative continuous ramp voltage recording of the Mrs2 channel activity recorded from −100 mV and +100 mV in the presence and absence of 5 mM Cobalt. (**i**) Displays the continuous recording of Mrs2 at −100mV and channel inhibition with added Cobalt (5 mM). (**j**) Group data for peak conductances of Mrs2 lipid bilayer recordings in response to Cobalt, unpaired t-test was used, P=0.002. All the channel recordings were performed in 150 mM NMDG and 10 mM HEPES solution buffered at (pH 7.4) and supplemented with 20–104 mM Mg^2+^ during recordings.

To confirm if matrix [Mg^2+^] regulates MCU channel activity, we recorded MCU Ca^2+^ currents (*I*_*MCU*_) in mitoplasts isolated from HeLa cells. We used varying [Mg^2+^] (0, 1, 5, and 10 mM) in the patch-clamp pipette solution that filled the mitochondrial matrix and measured *I*_*MCU*_. MCU currents were large with pipette solutions containing 0 and 1 mM Mg^2+^, whereas they were inhibited by ~30 and 65% at 5 and 10 mM Mg^2+^ (Fig. 1a and 1b). Having observed increasing pipette [Mg^2+^] to inhibit MCU activity (Fig. 1a and 1b) and Mg^2+^ to disrupt MCU homo-oligomerization^33^, we next asked whether increasing [Mg^2+^] also disrupts the hetero-oligomeric complex of MCU. We ectopically co-expressed MCU-HA with MICU1/MCUR1/EMRE/MCUb – Flag. MICU1/MCUR1/EMRE/MCUb co-immunoprecipitated with MCU-HA in 0 and 1 mM Mg^2+^. Increasing [Mg^2+^] disrupted the interaction of MCU with the positive regulators (MCUR1 and EMRE). In contrast, it preserved the interaction with negative regulators (MICU1 and MCUb) (Fig. 1c). Together, these results demonstrate that the mitochondrial matrix [Mg^2+^] regulates MCU activity by altering both the homo and hetero-oligomeric nature of MCU supercomplex (Fig.1a–1c).

Mitochondrial RNA splicing 2 (Mrs2) was initially considered the primary Mg^2+^ transporter in *Saccharomyces cerevisiae* mitochondria^35, 36^. The electrophysiological studies showed yeast Mrs2 (sMrs2) to form an Mg^2+^- selective channel (155 pS) and not permeable to Ca^2+ 36^. Though the core/pore component of the mMg^2+^ influx machinery was defined in yeast, the physiologic relevance of this observation in higher-order systems remains understudied. A recent study from Madesh and colleagues showed mammalian Mrs2 (mMrs2) to form a selective pore for lactate-mediated mMg^2+^ uptake^37^, authenticating Mrs2 as the conserved mMg^2+^ transport machinery from yeast to mammals. Though mMg^2+^ influx machinery was defined in mammals, the electrophysiological properties of mMrs2 are not established. To show whether mMrs2 forms an Mg^2+^-selective channel, we purified mMrs2 from HEK293 cells (Fig. 1d). The highly purified Mrs2 was used in planar lipid bilayer recordings for studying its single-channel activity. Our recordings show mMrs2 to form a voltagegated Mg^2+^ selective channel with multiple subconductance states (Fig. 1e–g). We recorded channels with the peak conductance activity varying from 80pS-250pS and mean conductance activity of ~150 pS (Fig. 1e–1j). Single-channel currents were recorded in the presence of Mg^2+^ only in Na^+^-, K^+^-, Cl^−^-, and Ca^2+^-free buffer and were shown to be inhibited by Co^2+^ (Fig. 1h–j). The continuous ramp voltage recording of the Mrs2 channel from −100 mV to +100 mV shows that the channel is inhibited by Co^2+^ at all voltages (Fig. 1h). Fig. 1i shows a continuous lipid bilayer recording of Mrs2 before and after adding Co^2+^.

**Figure 2.**
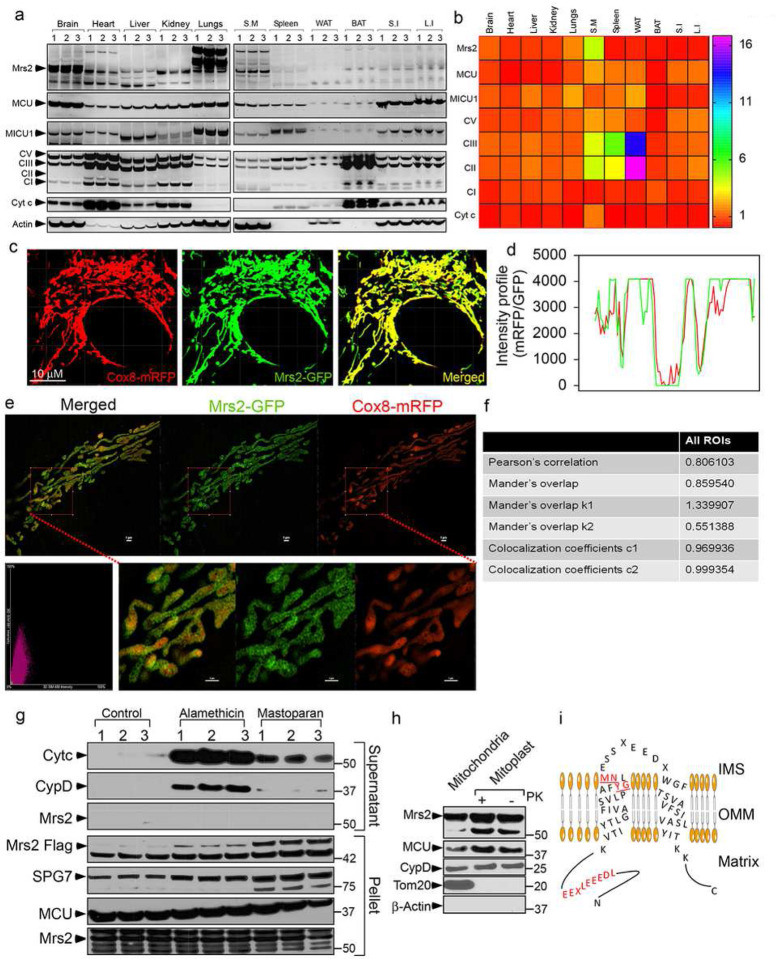
Mrs2, the integral membrane protein, localizes to the inner mitochondrial membrane with its N and C-termini in the matrix. (**a and b**) Representative Western blot (**a**) and densitometry quantification (**b**) show Mrs2 to be expressed ubiquitously in all metabolically active tissues. (**c and d**) Representative confocal images of HeLa cells transfected with Cox8-mRFP (Red) and Mrs2-GFP (Green) (**c**) and fluorescence intensity profile (**d**) show an overlap of RFP and GFP signals authenticating localization of Mrs2 to mitochondria. (**e**) Representative images of HeLa cells co-transfected with the Cox8-mRFP (Red)/Mrs2-GFP (Green) and imaged using SR-SIM. (**f**) Pearson, Manders, and overlap coefficients between Mrs2 and COX8-RFP substantiate a mitochondrial localization of Mrs2. (**g**) To assess the localization of Mrs2, HEK293 cells expressing Mrs2^WT^-Flag were permeabilized and exposed to mastoparan or alamethicin (20 μg/ml) for 5 min. Cytosolic (supernatant) and mitochondrial (pellet) fractions were subjected to immunoblotting to examine the release of cytochrome c (intermembrane space marker), CypD (matrix marker), Mrs2, SPG7, and MCU from mitochondria. Lanes 1, 2, and 3 refer to triplicate samples. Western blot shows the release of Cyt*C* and CypD after OMM/IMM permeabilization but not Mrs2, MCU, or SPG7, confirming Mrs2 as an integral membrane protein. (**h**) Immunoblot analyses of mitochondria and mitoplast from HEK293 cells expressing Mrs2^WT^-Flag. The freshly prepared mitoplasts were exposed to Proteinase K for 10 min. The samples were probed using antibodies specific for Flag, MCU, CypD, Tom20, and actin. Enrichment of Mrs2 in the mitoplasts with no loss of Flag tag suggests Mrs2 as an integral membrane protein that localizes to the IMM with its N and C-termini facing the matrix. (**i**) Schematic representation of Mrs2 with its functional domains in the N-terminus, Transmembrane, and IMS loop.

After confirming, Mrs2 channel activity, we extracted RNA and proteins from a panel of adult mouse tissues to verify whether Mrs2 is ubiquitously expressed in all mammalian tissues. Though differentially expressed, qPCR and Western blot analysis show, Mrs2 to be ubiquitously present in all metabolically active tissues (Fig. 2a and 2b; supplementary fig. 1a-1c). After confirming the ubiquitous distribution of Mrs2, we asked whether Mrs2 localizes explicitly to the mammalian mitochondria. To verify the mitochondrial distribution of Mrs2, we performed confocal microscopy and super-resolution structured illumination microscopy (SIM) imaging in HeLa cells expressing Mrs2-GFP and Cox8-mRFP. Confocal images at 488/561 nm and the intensity profile analysis overlap GFP and mRFP signals (Fig. 2c and 2d). Additionally, the SIM images and the correlation analysis substantiate a mitochondrial localization of Mrs2 (Fig. 2e and 2f). After confirming the localization of Mrs2 to the mitochondria, we performed a Western blot analysis to study the sub-organellar localization and orientation of Mrs2^12^. HEK 293 cells stably expressing Mrs2-Flag were permeabilized with digitonin (40 μg/ml). Digitonin-permeabilized cells were incubated with mastoparan or alamethicin to elicit outer or inner mitochondrial membrane permeabilization. OMM permeabilization (with mastoparan) was confirmed by the cytosolic appearance of cytochrome *C* (Fig. 2g). Permeabilization of both OMM and IMM (with alamethicin) was marked by the cytosolic appearance of cyclophilin D (matrix protein) (Fig. 2g). Mrs2 was observed in the membrane fraction in both mastoparan or alamethicin-treated cells, revealing Mrs2 as an integral membrane protein (Fig. 2g). The orientation of Mrs2 was evaluated by mitochondrial subfractionation^18^. Mitoplasts prepared from HEK293 cells stably expressing C-terminal Flag-tagged Mrs2 were subjected to proteinase K digestion followed by Western blot analysis with antibodies specific for Flag suggested Mrs2 to be enriched in mitoplasts (Fig. 2h). A mitochondrial matrix protein, CypD, was protected from proteinase K digestion (Fig. 2h). Similarly, the integral membrane protein Mrs2 remained stable with no loss of Flag-tag, suggesting the C-terminal end of Mrs2 to face the mitochondrial matrix. These results suggest that N and C termini face the mitochondrial matrix side, consistent with two transmembrane-spanning regions (Fig. 2i).

**Figure 3.**
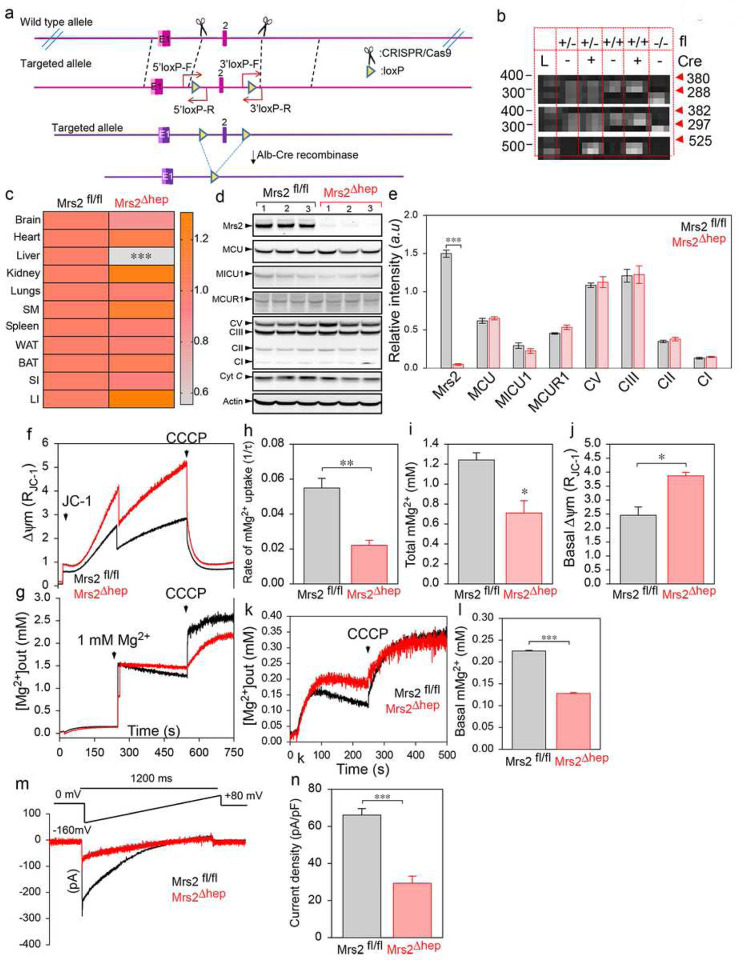
Loss of Mrs2 alters the mMg^2+^ uptake and the matrix [Mg^2+^]. (**a**) Schematic representation of loxP site insertion by CRISPR/Cas9 strategy and the generation of liver-specific Mrs2 KO (Mrs2^Δhep^) by CreloxP system. (**b**) PCR with redundant primers confirmed the genotype of control and KO mice. (**c**) Quantitative measurement of Mrs2 mRNA levels in different tissues collected from Mrs2^fl/fl^ and Mrs2^Δhep^ show liver-specific loss of Mrs2. (**d and e**) Representative Western blot (**d**) and densitometry quantification (**e**) show complete loss of Mrs2 in hepatocytes with no alterations to other mitochondrial proteins. (**f-j**) Simultaneous measurement of Δψm and mMg^2+^ uptake in hepatocytes isolated from Mrs2^fl/fl^ and Mrs2^Δhep^. Mean traces of Δψm (**f**) and [Mg^2+^]_out_ (**g**) in Mrs2^fl/fl^ (black) and Mrs2^Δhep^ (red). (**h**) Quantifying the rate of mMg^2+^ uptake as a function of the decrease in [Mg^2+^]_out_ after 1 mM Mg^2+^ pulse. (**i**) Quantification of the [Mg^2+^]_out_ after CCCP addition. (**j**) Quantification of the basal Δψm before adding 1 mM Mg^2+^ pulse. Data represents Mean ± SEM; *P<0.05, **P <0.01; n = 4. (**k**) Mean [Mg^2+^]_out_ traces before and after CCCP (5 μM) addition in Mrs2^fl/fl^ and Mrs2^Δhep^ hepatocytes. (**l**) Quantification of resting matrix [Mg^2+^] after the addition of CCCP. (**m and n**) Measurement of *I*_*Mrs2*_ current in mitoplasts prepared from Mrs2^fl/fl^ and Mrs2^Δhep^ hepatocytes. Traces are a single representative recording of *I*_*Mrs2*_. (**n**) *I*_*Mrs2*_ densities (pA/pF) in Mrs2^fl/fl^ and Mrs2^Δhep^. Bar represents Mean ± SEM; ***P < 0.001; n = 4–7.

Because we confirmed Mrs2 as the mMg2+ influx machinery, we anticipate losing Mrs2 to decrease matrix [Mg2+] and positively regulate MCU activity. To verify whether matrix [Mg2+] regulates MCU activity, we generated a liver-specific Mrs2 KO mouse model. Loxp/loxp knockin mice (Mrs2fl/fl) were generated using CRISPR/Cas9 strategy. Mrs2fl/fl mice were crossed with Albumin-Cre to allow germline deletion specifically in the liver (Mrs2Δhep) (Fig.3a). The gene targeting was confirmed by genotyping (Fig.3b). The loss of Mrs2 was confirmed using qPCR and Western blot analysis (Fig.3c–3e). The loss of Mrs2 did not alter the expression of other mitochondrial proteins like the OXPHOS complex, MCU, MICU1, and MCUR1 (Fig.3d–3e). We next asked whether loss of Mrs2 alters the mMg2+ uptake. Hepatocytes isolated from Mrs2fl/fl and Mrs2Δhep were permeabilized, and mMg2+ uptake and mitochondrial membrane potential (Δψm) were measured simultaneously (Fig. 3f and 3g) using a multi-wavelength-excitation dual wavelength-emission spectrofluorometer at 340/380 nm. The ratiometric dye, Mag-Fura-2 tetra potassium salt, was calibrated, and the mitochondrial [Mg2+] was determined by [Mg2+]=Kd((R−Rmin)/(Rmax−R)) where the dissociation constant Kd is 1.98 mM. Mrs2Δhep mitochondria showed no mMg2+ uptake in response to extramitochondrial Mg2+ pulse (Fig. 3g–3i). The loss of mMg2+ uptake was not due to changes in Δψm (Fig. 3f and 3j; supplementary Fig. 2a and 2b). We next asked whether the loss of Mrs2 caused a change in the total matrix [Mg2+] during the resting state. Digitonin permeabilized Mrs2fl/fl and Mrs2Δhep hepatocytes were loaded with Mag-Fura-2 tetra potassium salt. After baseline measurement of fluorescence, CCCP was added to depolarize the mitochondrial membrane (Fig. 3k). Loss of Mrs2 was marked by a decrease in the matrix [Mg2+] (Fig. 3k and 3l).

Because mitochondria can accumulate and release Mg^2+^ in the presence of stimuli, we then asked whether loss of Mrs2 alters cytosolic Mg^2+^ (cMg^2+^) dynamics. Mrs2^fl/fl^ and Mrs2^Δhep^ hepatocytes were loaded with MagGreen-AM and TMRM. cMg^2+^ and mMg^2+^ dynamics were measured simultaneously after stimulating the cells with 10 μM glucagon and 2 mM Mg^2+^ (Supplementary Fig. 2c and 2d). Confocal measurements show Mrs2^Δhep^ hepatocytes to have delayed cMg^2+^ clearance (Supplementary Fig. 2c-2g) compared to Mrs2^fl/fl^, confirming mitochondria play a critical role in regulating cellular Mg^2+^ dynamics. We performed mitoplast patch clamp recordings to verify whether loss of Mrs2 ablates mitochondrial Mg^2+^ current (*I*_*Mrs2*_). Loss of Mrs2 significantly ablated *I*_*Mrs2*_ validating Mrs2 as an authentic mammalian mMg^2+^ channel (Fig. 3m and 3n). Finally, to validate the *I*_*Mrs2*_ recordings, we performed mitoplast patch clamp recordings in Mrs2^fl/fl^ mitochondria in the presence of 200 nM ruthenium red (Ru360) and 1 μM hexamine cobalt(III)chloride. Hexamine cobalt(III)chloride, not Ru360, abolished *I*_*Mrs2*_, confirming the identity of *I*_*Mrs2*_ recordings as Mrs2 currents (Supplementary Fig. 2h-2i).

**Figure 4.**
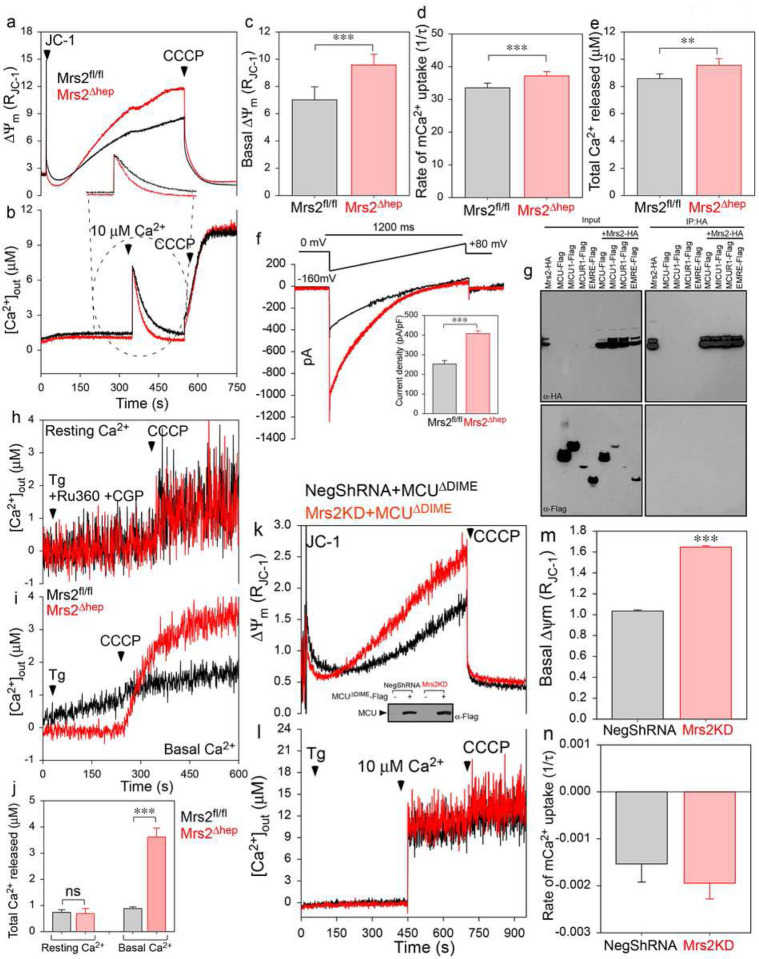
Loss of mMg^2+^ uptake potentiates MCU-mediated mCa^2+^ uptake. (**a-e**) Simultaneous measurement of Δψm and mCa^2+^ in hepatocytes isolated from Mrs2^fl/fl^ and Mrs2^Δhep^. Mean traces of Δψm (**a**) and [Ca^2+^]_out_ (**b**) in Mrs2^fl/fl^ (black) and Mrs2^Δhep^ (red). (**c**) Quantification of the basal Δψm before the addition of 10 μM Ca^2+^ pulse. (**d**) Quantification of the rate of mCa^2+^ uptake as a function of the decrease in [Ca^2+^]_out_ after 10 μM Ca^2+^ pulse. (**e**) Quantification of the [Ca^2+^]_out_ after CCCP addition. Data represent Mean ± SEM; **P<0.01, ***P <0.001; n = 4. (**f**) Measurement of *I*_*Mcu*_ current in mitoplasts prepared from Mrs2^fl/fl^ and Mrs2^Δhep^ hepatocytes. Traces are a single representative recording of *I*_*Mcu*_. (**inset**) *I*_*Mrs2*_ densities (pA/pF) in Mrs2^fl/fl^ and Mrs2^Δhep^. Bar represents Mean ± SEM; ***P < 0.001; n = 4–7. (**g**) HEK293 cells were co-transfected with HAtagged Mrs2 and Flag-tagged MCU, MICU1, MCUR1, and EMRE. Following immunoprecipitation with HA antibody, total cell lysates and immunoprecipitated materials were subjected to Western blot analysis. Cell lysates were probed with anti-HA (top left panel) or anti-Flag antibodies (bottom left panel) to serve as inputs. Immunoprecipitated samples were probed with anti-HA (top right panel) and anti-Flag (bottom right panel) antibodies (n=3). (**h and i**) Mean [Ca^2+^]_out_ traces before and after CCCP (5 μM) addition in Mrs2^fl/fl^ and Mrs2^Δhep^ hepatocytes in the presence (**h**) or absence (**i**) of Ru360 (MCU blocker) and CGP (NCLX blocker). (**j**) Quantification of resting and basal matrix [Ca^2+^] after adding CCCP. (**k-n**) Simultaneous measurement of Δψm and mCa^2+^ in HEK293 NegShRNA and Mrs2KD cells expressing MCU^ΔDIME^. Mean traces of Δψm (**k**) and [Ca^2+^]_out_ (**l**) in Mrs2^fl/fl^ (black) and Mrs2^Δhep^ (red). (**inset**) representative Western blot shows the expression of MCU^ΔDIME^ in HEK293 NegShRNA and Mrs2KD cells. (**m**) Quantification of the basal Δψm before adding 10 μM Ca^2+^ pulse. (**n**) Quantification of the rate of mCa^2+^ uptake as a function of the decrease in [Ca^2+^]_out_ after 10 μM Ca^2+^ pulse. Bar represents Mean ± SEM; ns non-significant; n = 4–7.

To validate the role of mMg^2+^ in regulating MCU-mediated mCa^2+^ uptake, we used our permeabilized cell system to perform simultaneous measurements of mCa^2+^ uptake and Δψ_m_^27, 38^ (Fig. 4a and 4b). A single bolus of extramitochondrial Ca^2+^ pulse (10 μM) was added, and the rate of mCa^2+^ uptake was measured as a function of the decrease in bath Ca^2+^ fluorescence. Mrs2^Δhep^ cells could rapidly clear extramitochondrial Ca^2+^ pulse, indicating increased MCU activity (Fig. 4a–4e). Mitoplast patch-clamp recordings confirmed increased MCU activity (*I*_*MCU*_) in Mrs2^Δhep^ mitoplasts (Fig. 4f and inset). Next, we asked whether Mrs2 binds directly to any of the MCU complex components and increases the MCU activity or is an indirect regulation through altering matrix [Mg^2+^]. The immunoprecipitation with HA antibody could pull down Mrs2 (Fig. 4g; top right) with no notable interaction of Mrs2 with MCU, MICU1, MCUR1, and EMRE (Fig. 4g; bottom right), confirming no direct interaction between Mrs2 and other MCU complex components. Not just in hepatocytes, we also observed loss of Mrs2 (Supplementary Fig. 3a-3c) and decreased mMg^2+^ uptake (Supplementary Fig. 3d and 3e) in HEK293 cells to alter MCU activity (Supplementary Fig. 3f-3i). Previously we showed the binding of Mg^2+^ to the MRAP region to destabilize and shift the self-association equilibrium of MCU to monomer^33^. Consistent with our previous finding, FPLC analysis showed a heterogenous population of MCU complex (both low and high molecular weight) in HEK 293 NegShRNA cells, whereas MCU assembled as a supercomplex in Mrs2 KD cells (Supplementary Fig. 3j and 3k). Collectively, these results suggest the loss of mMg^2+^ to stabilize the MCU complex and promote channel activity.

We next asked whether increased MCU activity overloads matrix Ca^2+^ at the resting state. Permeabilized cells loaded with Fura-FF tetra potassium salt were treated with Ru360 (to block any extramitochondrial Ca^2+^ uptake) and CGP (to block the efflux of mitochondrial Ca^2+^). After baseline measurement, an uncoupler CCCP was added to depolarize the mitochondrial membrane (Fig. 4h). Though MCU activity was increased in Mrs2^Δhep^, we do not observe an increase in resting matrix [Ca^2+^] (Fig. 4h and 4j). Interestingly, when Ru360 was removed from the experimental condition described above, we observed the matrix [Ca^2+^] significantly higher in Mrs2^Δhep^ than Mrs2^fl/fl^ (Fig. 4i and 4j). Because the assay buffer contains Thapsigargin (Tg), we anticipated the loss of Mrs2 and the decreased matrix [Mg^2+^] concentration to activate MCU in the low-[iCa^2+^] regime^39^. We used our permeabilized cell system to simultaneously measure mCa^2+^ uptake and Δψm (Supplementary Fig. 4a-4i). Mrs2^Δhep^ rapidly cleared the 1 μM Ca^2+^ pulse, whereas the channel gatekeeping function remained intact in Mrs2^fl/fl^ (Supplementary Fig. 4b, 4d-4e). Additionally, the bath Ca^2+^ before adding Ca^2+^ pulse was lower in Mrs2^Δhep^ compared to Mrs2^fl/fl^ (Supplementary Fig. 4f and 4g). Also, the bath Ca^2+^ after CCCP addition was significantly higher in Mrs2^Δhep^ than Mrs2^fl/fl^ (Supplementary Fig. 4h and 4i), confirming the loss of Mrs2 to activate MCU in the low-[iCa^2+^] regime. We asked whether the loss of Mrs2 alters MCU/MICU1 interaction to activate MCU in the low-[iCa^2+^] regime. The immunoprecipitation with Flag antibody pulled down MCU and MICU1, indicating MCU/MICU1 complex to be intact in Mrs2 KD cells (Supplementary Fig. 4j).

Since we saw increased MCU activity in the low-[Ca^2+^]i regime despite stable MCU/MICU1 interaction, we asked whether the increased mCa^2+^ uptake is strictly MCU-mediated. We stably expressed MCU^ΔDIME^ mutant in HEK293 NegShRNA and Mrs2KD cells. MCU^ΔDIME^ expression significantly decreased MCU-mediated mCa^2+^ uptake in both control and KD cells (Fig. 4k–4n), authenticating the regulation of MCU-channel activity by matrix [Mg^2+^]. Though MICUs sets a [iCa^2+^] threshold for MCU activation, our results indicate that the regulation of MCU is also to be governed by the matrix [Mg^2+^] concentration, reinforcing the concept of Mg^2+^dependent MCU regulation.

**Figure 5.**
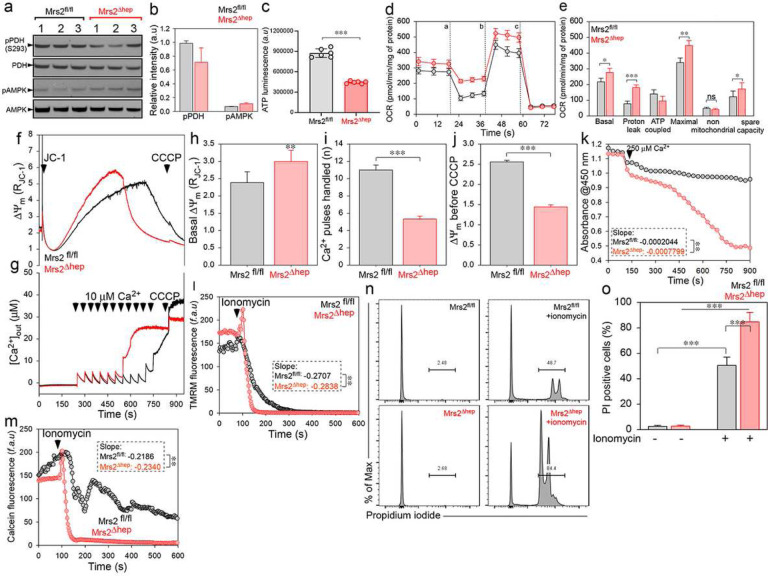
Loss of mMg^2+^ uptake alters the cellular bioenergetics and induces Ca^2+^ overload-mediated PTP opening. (**a and b**) Representative Western blot was probed with antibodies specific for phospho PDH and phospho AMPK. The phosphorylation of PDH and AMPK was normalized with the total PDH or AMPK. (**b**) Densitometry quantification show decreased and increased phosphorylation of PDH and AMPK, respectively. (**c**) Quantifying total cellular ATP levels in Mrs2^fl/fl^ and Mrs2^Δhep^ hepatocytes. (**d**) Mean traces of oxygen consumption rate (OCR) in Mrs2^fl/fl^ and Mrs2^Δhep^ hepatocytes using pyruvate as a substrate and after sequential exposure to Oligomycin (a), FCCP (b), and rotenone/antimycin (c). (**e**) Quantification of basal, maximal OCR, proton leak, ATP coupled respiration, spare capacity, and non-mitochondrial respiration. Data indicate Mean ± SEM; *P < 0.05, **P < 0.01, ***P < 0.001; n = 4–10. (**f-j**) Simultaneous measurement of Δψm and mCa^2+^ in Mrs2^fl/fl^ and Mrs2^Δhep^ hepatocytes. Mean traces of Δψm (**f**) and [Ca^2+^]_out_ (**g**) in Mrs2^fl/fl^ (black) and Mrs2^Δhep^ (red) after a series of 10 μM Ca^2+^ pulse. (**h**) Quantifying the basal Δψm before adding the first 10 μM Ca^2+^ pulse. (**i**) Quantification of the number of Ca^2+^ pulses handled before the loss of Δψm. (**j**) Quantification of the Δψm before the addition of CCCP. Bar represents Mean ± SEM; **P < 0.01, ***P < 0.001; n = 4–7. (**k**) Isolated mitochondria from Mrs2^fl/fl^ and Mrs2^Δhep^ hepatocytes were incubated with Ca^2+^ (250 μM), and mitochondrial swelling was measured as absorbance at 540 nm. The mean value traces were plotted with a polynomial fit. n=3–6. (**l**) Mean traces of TMRM fluorescence in Mrs2^fl/fl^ and Mrs2^Δhep^ hepatocytes before and after adding ionomycin (5 μM). (**m**) Mean traces of mitochondrial calcein fluorescence in Mrs2^fl/fl^ and Mrs2^Δhep^ hepatocytes before and after the addition of ionomycin (5 μM). (**n and o**) Mrs2^fl/fl^ and Mrs2^Δhep^ hepatocytes were treated with ionomycin (5 μM) for 6 h, stained with propidium iodide, and cell death was measured by flow cytometry. Representative histogram (**n**) and quantification of PI-positive cells (**o**) show increased cell death in Mrs2^Δhep^ hepatocytes. Mean ± SEM; ***p<0.001, n=3–4.

Pyruvate dehydrogenase (PDH) is the enzyme that catalyzes the irreversible conversion of pyruvate to acetyl-CoA. PDH is regulated through the alterations in the phosphorylation state of the enzyme by PDH kinase (PDHK) and PDH phosphatase (PDHP). The major regulators of PDHP are the divalent cations Mg^2+^ and Ca^2+^. mMg^2+^ has been documented to enhance the activity of PDHP^40^. PDHP is inactive in the absence of Mg^2+^ and can be activated 10–20 fold by Ca^2+^ only in the presence of Mg^2+^. Because we saw decreased and increased matrix [Mg^2+^] and [Ca^2+^], we next asked whether the loss of Mrs2 affects the phosphorylation state of PDH. Though insignificant, we saw a trend in the decreased PDH phosphorylation in Mrs2^Δhep^ compared to Mrs2^fl/fl^ (Fig. 5a and 5b). The decrease in PDH phosphorylation can be attributed to increased matrix [Ca^2+^], but the loss in complete PDH dephosphorylation marks the critical need for matrix Mg^2+^ to activate PDHP. Because we saw a trend in decreased PDH phosphorylation, we assessed the ATP levels in control and KO/KD cells. ATP was significantly decreased in KO/KD cells compared to control (Fig. 5c, Supplementary Fig. 5a). The decrease in ATP levels was marked by an increase (not significant) in AMPK phosphorylation (Fig. 5a and 5b).

Because we saw opposing results with PDH activation and ATP levels, we assessed the oxygen consumption rate (OCR) in control and Mrs2 KO/KD cells (Fig. 5d, 5e, Supplementary Fig. 5b, and 5c). Basal and maximal respiration was increased in KO/KD cells compared to the control (Fig. 5d, 5e, Supplementary Fig. 5b, and 5c). Further analysis of the OCR data showed increased proton leak in KO/KD cells with a concomitant decrease in ATP-coupled respiration (Fig. 5d, 5e, Supplementary Fig. 5b, and 5c). Though the proton leak was increased in Mrs2 KO/KD cells, we do not see a decrease in Δψm. Instead, a significant increase in Δψm was observed (Fig. 3f, 3j, 4a, 4c, 5f, 5h, supplementary fig. 2a and 2b, supplementary fig. 4a and 4b), ruling out the possible contribution of uncoupling proteins (UCP) to proton leak.

According to the chemiosmotic theory, the Δψm can be maintained either by the electron transport chain through respiration or by ATP hydrolysis via the F1-F0 ATPase. Because we observed increased proton leak and decreased ATP, we asked whether the reversal of F1-F0 ATPase contributes to increased Δψm. Using our permeabilized cell system, we measured Δψm in Mrs2^fl/fl^ and Mrs2^Δhep^ in the presence or absence of Oligomycin (ATP synthase and hydrolysis inhibitor) or BTB06584 (selective inhibitor of ATP hydrolysis). Both Oligomycin and BTB treatment depolarized/normalized the Δψm in Mrs2^Δhep^. Conversely, oligomycin treatment hyperpolarized the Mrs2^fl/fl^ mitochondria, and BTB had no effect (Supplementary Fig. 5d and 5e). Because the reversal of ATP synthase depletes cellular ATP and activates the mitochondrial permeability transition pore (mPTP)^41^, we next asked whether increased matrix Ca^2+^ overload and ATP reversal make Mrs2^Δhep^ susceptible to mPTP opening. We simultaneously measured calcium retention capacity (CRC) and Δψm in Mrs2^fl/fl^ and Mrs2^Δhep^. Mrs2^Δhep^ exhibited decreased CRC associated with an early Δψm collapse (Fig. 5f–5j), indicating matrix Ca^2+^ overload-induced activation of the mPTP. Increased Ca^2+^-induced mitochondrial swelling (Fig. 5k), early TMRM/calcein fluorescence loss (Fig. 5l and 5m), and increased ionomycin-mediated cell death confirmed matrix Ca^2+^ overload-induced mPTP opening in Mrs2^Δhep^ (Fig. 5n and 5o).

**Figure 6.**
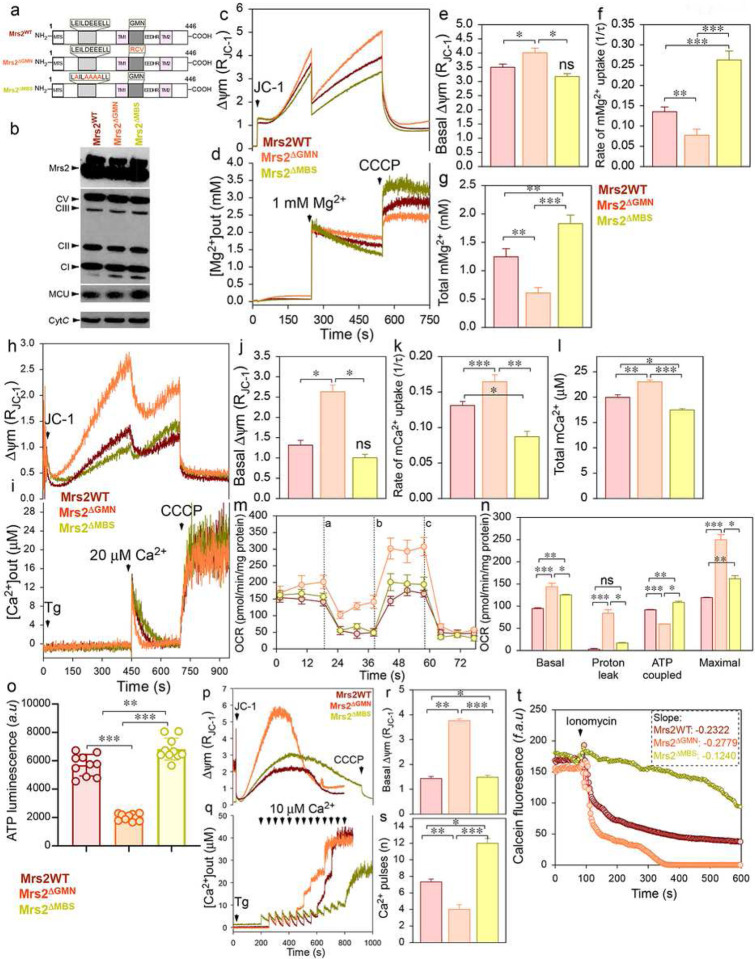
Mrs2 gain-of-function mutant augments mMg^2+^ uptake and prevents Ca^2+^-overload mediated PTP opening. (**a**) Schematic of full-length Mrs2 with its functional domains (Mrs2^WT^). Mutations in Mrs2^WT^ are highlighted in red (Mrs2^ΔGMN^ and Mrs2^ΔMBS^). (**b**) Representative Western blot probed with antibodies specific for Flag shows the expression of Mrs2^WT^, Mrs2^ΔGMN^, and Mrs2^ΔMBS^ in HEK293 WT cells. (**c-g**) Simultaneous measurement of Δψm and mMg^2+^ in HEK 293 cells expressing Mrs2^WT^, Mrs2^ΔGMN^, and Mrs2^ΔMBS^. Mean traces of Δψm (**c**) and [Mg^2+^]_out_ (**d**). (**e**) Quantification of the basal Δψm before the addition of 1 mM Mg^2+^ pulse. (**f**) Quantifying the rate of mMg^2+^ uptake as a function of the decrease in [Mg^2+^]_out_ after 1 mM Mg^2+^ pulse. (**g**) Quantification of the [Mg^2+^]_out_ after CCCP addition. Data represents Mean ± SEM; *P<0.05, **P <0.01; n = 4. (**h-l**) Simultaneous measurement of Δψm and mCa^2+^ in HEK 293 cells expressing Mrs2^WT^, Mrs2^ΔGMN^, and Mrs2^ΔMBS^. Mean traces of Δψm (**h**) and [Ca^2+^]_out_ (**i**). (**j**) Quantification of the basal Δψm before adding 20 μM Ca^2+^ pulse. (**k**) Quantification of the rate of mCa^2+^ uptake as a function of the decrease in [Ca^2+^]_out_ after 20 μM Ca^2+^ pulse. (**l**) Quantification of the [Ca^2+^]_out_ after CCCP addition. Data represent Mean ± SEM; **P<0.01, ***P <0.001; n = 4. (**m**) Mean traces of oxygen consumption rate (OCR) in HEK 293 cells expressing Mrs2^WT^, Mrs2^ΔGMN^, and Mrs2^ΔMBS^ using pyruvate as a substrate and after sequential exposure to Oligomycin (a), FCCP (b), and rotenone/antimycin (c). (**n**) Quantification of basal, maximal OCR, proton leak, and ATP-coupled respiration. Data indicate Mean ± SEM; *P < 0.05, **P < 0.01, ***P < 0.001; n = 10. (**o**) Quantification of total cellular ATP levels in HEK 293 cells expressing Mrs2^WT^, Mrs2^ΔGMN^, and Mrs2^ΔMBS^. (**p-s**) Simultaneous measurement of Δψm and mCa^2+^ in HEK 293 cells expressing Mrs2^WT^, Mrs2^ΔGMN^, and Mrs2^ΔMBS^. Mean traces of Δψm (**p**) and [Ca^2+^]_out_ (**q**) after a series of 10 μM Ca^2+^ pulses. (**r**) Quantification of the basal Δψm before adding the first 10 μM Ca^2+^ pulse. (**s**) Quantification of the number of Ca^2+^ pulses handled before the loss of Δψm. Bar represents Mean ± SEM; **P < 0.01, ***P < 0.001; n = 4–7. (**t**) Mean traces of mitochondrial calcein fluorescence in HEK 293 cells expressing Mrs2^WT^, Mrs2^ΔGMN^, and Mrs2^ΔMBS^ before and after adding ionomycin (5 μM).

Next, we used gain/loss-of-function Mrs2 mutants to examine whether the increased MCU activity and matrix Ca^2+^ overload-induced activation of the mPTP are causal effects of decreased mMg^2+^ uptake. It has been proposed that single amino acid substitutions in the G-M-N motif of Mrs2 are sufficient to abolish Mg^2+^ transport or profoundly change the ion selectivity of channel^42–44^. Additionally, reports had shown eventually complete inhibition of CorA-driven Mg^2+^ currents when the intracellular domain of CorA was exposed to mM concentration of Mg^2+ 45, 46^. This phenomenon could be due to a self-regulatory mechanism, where an increase in the local [Mg^2+^] saturates the putative Mg^2+^ binding site (MBS), triggering channel closure^46^. We generated loss-of-function (Mrs2^ΔGMN^) and gain-of-function (Mrs2^ΔMBS^) mutant constructs (Fig. 6a). Western blot confirms the ectopic expression of Flag-tagged Mrs2^WT^, Mrs2^ΔGMN^, and Mrs2^ΔMBS^ in HEK293 WT cells (Fig. 6b). The permeabilized cell system analysis (Fig. 6c–6g) show abolished and increased mMg^2+^ uptake in Mrs2^ΔGMN^ and Mrs2^ΔMBS^, respectively (Fig. 6d, 6f, and 6g). The expression of Mrs2^WT^ did not alter the mMg^2+^ uptake, further authenticating mMg^2+^ homeostasis to be maintained through a negative feedback loop^45, 46^. We next sought to define whether altered mMg^2+^ uptake modifies mCa^2+^ uptake. Simultaneous measurements of Δψm and mCa^2+^ (Fig. 6h–6l) show increased and decreased mCa^2+^ uptake in Mrs2^ΔGMN^ and Mrs2^ΔMBS^, respectively (Fig. 6i, 6k, and 6l). Similar to Mrs2KO/KD cells, basal and maximal OCR was increased in Mrs2^ΔGMN^ (Fig. 6m and 6n) with a decrease in ATP levels (Fig. 6o). The decrease in ATP levels and increased Δψm was due to increased proton leak (Fig. 6c, 6e, 6h, 6j, and 6n), further confirming the loss of mMg^2+^ to result in ATP synthase reversal. Our results also show decreased CRC (Fig. 6p–6s) and early calcein quenching (Fig. 6t) in Mrs2^ΔGMN^. The expression of Mrs2^ΔMBS^ preserved the mitochondrial function (Fig. 6m–6o) and delayed the Ca^2+^-mediated mPTP opening (Fig. 6p–6t). These data show MCU activity and Ca^2+^-mediated mPTP opening to be fine-tuned by Mrs2-mediated mMg^2+^ uptake.

**Figure 7.**
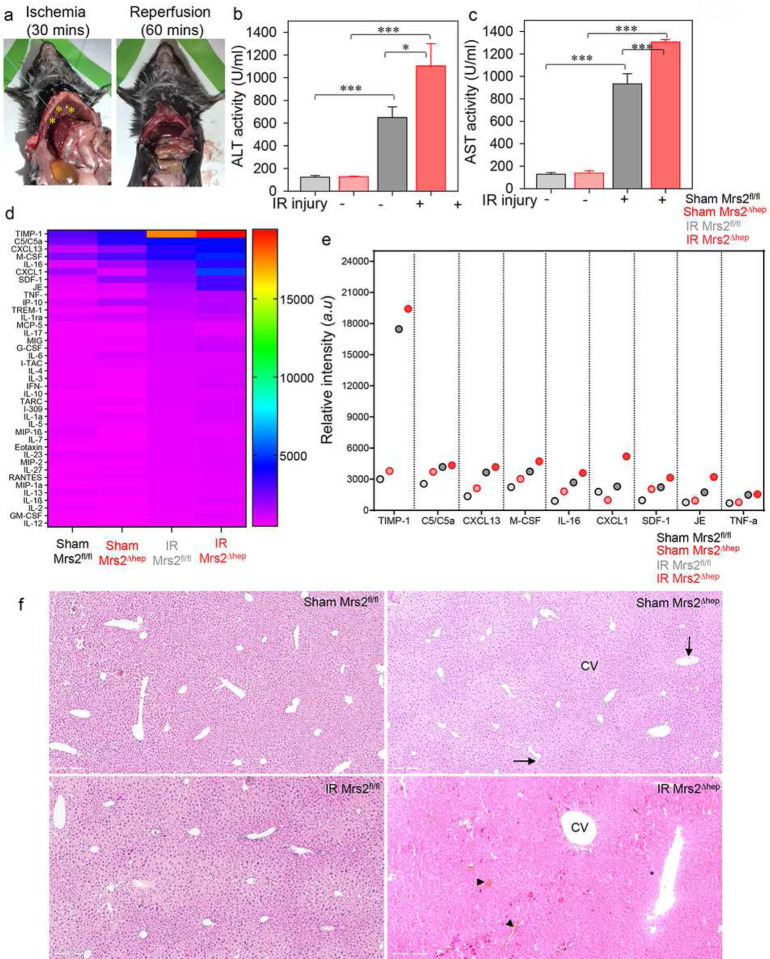
Loss of Mrs2 augments ischemic liver injury. (**a and b**) Model of ischemic liver reperfusion using the hanging-weight system. (**a**) Successful occlusion was confirmed by a change of color from red to pale (left panel; marked with a yellow asterisk), and the change of color immediately disappeared when the hanging weights were removed, and the liver was reperfused (right panel). (**b and c**) The ALT (b) and AST (c) were quantified in the sham and IR injured control and KO mice plasma. Data represent Mean ± SEM; *P<0.05, ***P <0.001; n = 4. (**d**) Densitometry quantification of the cytokine array and the representative heat map show increased levels of inflammatory cytokines in the plasma of Mrs2^Δhep^ compared to sham and Mrs2^fl/fl^ IR injured mice. (**e**) Quantification of the inflammatory cytokines significantly altered in the serum of Mrs2^Δhep^ compared to sham and Mrs2^fl/fl^ IR injured mice. The colored circle represents the Mean; *P<0.05, ***P <0.001; n = 4. (**f**) Histological analysis shows hepatocyte liver necrosis (marked by a black asterisk), mononucleated cell infiltration (arrow), enlarged central vein (cv), and increased congestion (arrowhead) in Mrs2^Δhep^ compared to sham and Mrs2^fl/fl^ IR injured mice.

To further confirm whether loss of Mrs2 potentiates MCU-mediated mCa^2+^ uptake, overloads matrix Ca^2+^, and induces cell death, we performed an acute liver ischemic reperfusion (IR) injury by hanging weight method. Using this method, we saw the portal triad immediately occluded by hanging the weights over the poles, causing the blood supply to the left, median, and caudate lobes of the liver to be interrupted. Successful occlusion was confirmed by visual inspection of pale blanching in the ischemic lobes (i.e., a change in color from red to a pale color) (Fig. 7a; left panel). In contrast, the change of color immediately disappeared when the hanging weights were removed from the poles, and the liver was reperfused (Fig. 7a, right panel). Because elevated plasma ALT and AST are reliable markers for liver parenchymal cell membrane integrity and liver injury^47, 48^, we measured ALT and AST activity in plasma after 30 mins of ischemia followed by 1 h of reperfusion. The ALT and AST levels were increased in Mrs2^Δhep^ (Fig. 7b and 7c). Our results also show increased levels of inflammatory cytokines in the plasma of Mrs2^Δhep^ (Fig. 7d and 7e) compared to sham and Mrs2^fl/fl^ IR injured mice. Histological analysis shows hepatocyte liver necrosis, mononucleated cell infiltration, enlarged central vein (cv), and increased congestion in Mrs2^Δhep^ compared to sham and Mrs2^fl/fl^ IR injured mice (Fig. 7f). Taken together; these data show Mrs2-mediated mitochondrial Mg^2+^ uptake is critical in maintaining MCU activity and protecting mitochondria from Ca^2+^ overload mediated mPTP opening during IR injury.

## DISCUSSION

Next to potassium (K^+^), magnesium (Mg^2+^) is the abundant intracellular divalent cation. It is an essential co-factor in the machinery replicating, transcribing, and translating genomic information^49–52^. As a structural cofactor, Mg^2+^ stabilizes the ribosome, lipid membranes, and nucleic acids^49–51, 53^. It is pivotal in metabolic networks and signaling cascades where it regulates enzyme activity, especially those requiring ATP^54–56^. Significantly Mg^2+^ alters the electrophysiological properties of ion channels^55^ and can also affect the binding affinity of Ca^2+^ to specific Ca^2+^- binding proteins^57,58,59,60, 61^, thus altering the iCa^2+^ dynamics and signaling^62^. Most cell types’ total [iMg^2+^] is ~20 mM^63, 64^. The free [Mg^2+^] is typically in the range of ~0.5–1.0 mM, and a considerable proportion of the iMg^2+^ pool exists as ATP-bound Mg^2+ 63, 64^. Remarkably, the free [iMg^2+^] is 100 fold below the electrochemical potential, theoretically indicating a tight regulation of iMg^2+^ entry^37^. Regulation of iMg^2+^ homeostasis occurs via numerous Mg^2+^ transport machinery on the plasma membrane, including the ubiquitous MagT1, ACDP, TRPM7, SLC41A1, and tissue-specific TRPM6, as well as cyclin M2 and cyclin M4^65–69,70–79^. Though these findings have enhanced the knowledge of the occurrence of significant Mg^2+^ fluxes in either direction across the plasma membrane of mammalian cells following metabolic or hormonal stimuli, little is known about the organellar mobilization of Mg^2+^. Nuclei, mitochondria, and endoplasmic or sarcoplasmic reticulum (ER/SR) compartmentalize iMg^2+^.

Mitochondrial Mg^2+^ (mMg^2+^) is known to significantly impact the metabolic state^80–93^, mitochondrial Ca^2+^ (mCa^2+^) homeostasis^94–97^, and apoptosis^98^. Mitochondria can accumulate and release Mg^2+^ in response to metabolic stimuli, thus representing a critical iMg^2+^ store^37, 99, 100^. Mitochondrial RNA splicing 2 (Mrs2) was identified as the primary Mg^2+^ transporter in yeast mitochondria^35, 36^ and formed a selective pore for lactatemediated mMg^2+^ uptake in mammals^39^, authenticating Mrs2 as the conserved mMg^2+^ transport machinery from yeast to mammals. Though Mrs2 has been shown as the transport machinery of mammalian mitochondria, Mrs2 has not been characterized. Here using the purified mammalian Mrs2 protein and the state-of-the-art planar lipid bilayer electrophysiological measurements, we characterized Mrs2 as the authentic mammalian mitochondrial Mg^2+^ channel (Fig. 1). Using biochemical assays, we show Mrs2 to be an integral membrane protein that localizes to the mitochondrial inner membrane with its N and C-termini within the matrix (Fig. 2). Recent structural analysis of the N-terminal domain of Mrs2 identified a novel mechanism by which Mrs2 is autoregulated by matrix [Mg^2+^] concentration^46^, implicating the N-terminal domain (NTD) to be critical for Mrs2 regulation and function.

Mitochondria display two different profiles of Ca^2+^ transients; a fast uptake of Ca^2+^ followed by a slower and more gradual uptake^97, 101–108^. Studies show increasing extramitochondrial Mg^2+^ ([Mg^2+^]_out_) to have a differential effect on these two modes of mCa^2+^ transients. Early initial velocity studies suggest Mg^2+^ be a competitive inhibitor for mCa^2+^ uptake where it induces sigmoid kinetics, and the degree of sigmoidicity increases with an increase in [Mg^2+^]_out_ without altering the maximal activity of the uniporter^97, 101–108^. However, subsequent studies on kinetics reported a non-competitive inhibition of mCa^2+^ uptake by [Mg^2+^]_out_ and are known to decrease the maximal activity of the uniporter^97, 101–108^. To define the mechanism by which Mg^2+^ regulates mCa^2+^ uptake, we previously resolved the atomic structure of the conserved N-terminal domain of MCU^33^. The crystal structure of the N-terminal domain revealed a β-grasp-like fold containing the MCU regulating acidic patch (MRAP)^33^. MRAP binds Mg^2+^/Ca^2+^ with ~mM affinity. The binding of divalent cations to MRAP destabilizes MCU, shifts the self-association equilibrium to monomer, and attenuates MCU-mediated mCa^2+^ uptake^33^. We anticipate divalent cations to regulate MCU activity based on our previous study.

Previously regulation of MCU channel activity was defined by the cooperative activation of MICU1/2 that sets a cytosolic Ca^2+^ threshold for MCU activity^13, 19, 39, 109–111^. However, recent seminal work showed that the regulation of MCU activity is set not only by the Ca^2+^ affinities of the MICU1/2 EF hands but also by matrix [Ca^2+^] and buffering capacity, allowing for enhanced regulation of mCa^2+^ homeostasis^34^. It is known that matrix [Ca^2+^] regulates MCU channel activity with a biphasic concentration dependence, with potent inhibition at ~100 nM and maximal channel inhibition (~80%) at ~400 nM^34^. Interestingly, high BAPTA (5 mM) and low matrix [Ca^2+^] also inhibit MCU activity^34^. If MCU is regulated by matrix [Ca^2+^], this result was unpredicted as the matrix Ca^2+^ regulatory sites should be unoccupied, and MICU1/2 should be fully Ca^2+^-liganded under the *I*_*MCU*_ record conditions, which together would be expected to activate the channel. Nevertheless, channel inhibition was observed.

To increase the electrical stability of the mitoplast, the authors used 2 mM MgCl_2_ in the pipette solution in all their *I*_*MCU*_ current measurements. Remarkably removal of Mg^2+^ from the pipette solution completely abolished MCU channel inhibition observed in high-[BAPTA]/low matrix [Ca^2+^] and also in 400 nM Ca^2+^/1.5 mM EGTA. Therefore, Mg^2+^ binding to the MRAP region might underlie channel inhibition observed in high-[BAPTA]/low [Ca^2+^]m. Though the possible role of matrix [Mg^2+^] in regulating MCU activity was proposed in earlier studies^34^, the molecular mechanism was not explored in detail. To understand the regulation of MCU by matrix [Mg^2+^], we generated a liver-specific Mrs2 KO mouse model (Fig. 3). The loss of Mrs2 drastically decreased the matrix [Mg^2+^] and thus would serve as an ideal model to study MCU regulation. Our results show a loss of Mrs2 to increase the MCU-mediated mCa^2+^ uptake. We measured the resting matrix [Ca^2+^] in control and Mrs2 KO cells. We observed no difference in the resting [Ca^2+^] in the presence of Ru360 and CGP, whereas the absence of both Ru360 and CGP drastically increased the resting matrix [Ca^2+^] (Fig.4). The increase in the matrix [Ca^2+^] shows the loss of Mg^2+^-dependent regulation of MCU. Because we saw an increased matrix [Ca^2+^] just by adding Tg to the bath, we anticipate the loss of Mrs2 to alter the iCa^2+^ threshold required to activate MCU. Also, we observed the interaction of MCU/MICU1 to be intact in the Mrs2 KD cells. Further, studies are warranted to understand the MICU1-dependent gating of MCU in the absence of matrix [Mg^2+^].

Because Ca^2+^ activates the mitochondrial permeability transition pore, we asked whether loss of MCU regulation in Mrs2 KO cells results in early mPTP opening. We observed early mPTP opening in Mrs2 KO cells (Fig. 5), similar to that observed in HEK cells in which the matrix Ca^2+^-dependent MCU regulation is abolished^34^. Our results also show Mrs2 KO mice susceptible to liver ischemia-reperfusion (IR) injury (Fig. 7). We anticipate the loss of Mrs2 to activate mPTP through two modes, direct and indirect regulation. Our results show that loss of Mrs2 positively regulates MCU activity, overloads matrix Ca^2+^, and triggers mPTP opening. However, Mg^2+^ is an inhibitor of mPTP opening^112^. Mg^2+^ modulates the mPTP pore’s sensitivity to cyclosporine A and ADP^113^. Additionally, work from the Bernardi laboratory show that the mPTP channel forms from dimers of F-ATP synthase after a conformational change that would follow a replacement of Mg^2+^ with Ca^2+^ at the catalytic site. Also, when Ca^2+^ replaces Mg^2+^, ATP hydrolysis is not coupled to forming an H^+^ gradient^114^.

Interestingly, our data show Mrs2 KO to have decreased ATP levels despite hyperpolarized mitochondria, suggesting an uncoupling of chemical catalysis from H^+^ translocation and H^+^ backflow through the mPTP channel (Supplementary Fig. 5). We also anticipate a fall in the electrochemical proton gradient and membrane potential depolarization in Mrs2 KO hepatocytes during early stages of development. The compensatory pathways induced by the loss of Mrs2 during development possibly reversed the direction of ATP synthase rotation, resulting in ATP hydrolysis. We anticipate the reversal of ATP synthase to extrude protons into the intermembrane space and contribute to restoring the proton gradient and the membrane potential while simultaneously resulting in a net loss of ATP^115, 116^. We used Oligomycin and BTB treatment to show the reverse activity of ATP synthase (Supplementary Fig. 5). Oligomycin blocks ATP hydrolysis and synthesis, whereas BTB is a specific ATP hydrolysis inhibitor. If the respiratory chain can keep up the membrane potential intact, then oligomycin treatment will hyperpolarize the mitochondria; however, if membrane potential is maintained by ATP hydrolysis, oligomycin/BTB will induce depolarization, a phenomenon termed as “oligomycin null-point”^117–119^. ATP hydrolysis can occur in extreme pathological conditions or normal or mildly compromised mitochondria, such as in humans with mitochondrial genetic disease and myopathies^120^. Our results demonstrate oligomycin null-point in Mrs2 KO mitochondria (Supplementary Fig. 5). We show in Mrs2 KO mitochondrial membrane potential to be maintained by the reversal of ATP synthase, thus depleting cellular ATP and activating mPTP opening^121^. We anticipate the increased matrix [Ca^2+^] to replace the available Mg^2+^ bound to the F1-F0 ATP synthase, inciting a conformational change in the functional protein dimer to the PTP^114^. Future studies will be necessary to understand the regulation of mPTP by loss of matrix [Mg^2+^] and the reversal of ATP synthase.

Our results show expression of gain-of-function Mrs2 mutant to protect cells from mPTP opening (Fig. 6). From our structural insight, we predict that at the physiological range of matrix [Mg^2+^], ~33 – 60% of MRAP will be occupied. Therefore, even at the highest non-physiological matrix [Mg^2+^], we do not anticipate a complete loss of MCU activity, thus maintaining the physiological MCU activity and energy homeostasis during pathology. Also, we show that Mrs2-mediated mitochondrial Mg^2+^ uptake is a critical regulator to prevent MCUmediated Ca^2+^ overload during I/R injury. However, future studies must study how MCU-mediated Ca^2+^ overload occurs during I/R injury, despite Mrs2-dependent MCU regulation in reality. We anticipate the loss of Mrs2 (either decreased channel density or decreased activity) to relieve MCU from Mg^2+^-dependent negative feedback regulation and increase MCU-mediated Ca^2+^ overload during disease pathology.

## CONCLUSION

In conclusion, our results show that regulating MCU by matrix [Mg^2+^] is pivotal in preserving mitochondrial function and facilitating adaptation to increased workload and disease states. Thus, changes in the free matrix [Mg^2+^] due to mitochondrial Mg^2+^ entry or changes in matrix phosphate, ATP, or ADP levels, the MRAP domain of MCU will sense these differences and regulate MCU channel activity.

## MATERIALS AND METHODS

### Animals:

Lox/loxp knockin mice (Mrs2^fl/fl^) with loxP sites flanking the exon 2 (ENSMUSE00000251120) were generated using CRISPR/Cas9 strategy. Next, Mrs2^fl/fl^ mice were crossed with liver-specific-Cre transgenic mice, albumin-Cre (B6.Cg-Speer6-ps1^Tg(Alb-cre)21Mgn/^J) to generate liver-specific Mrs2 knockouts (Mrs2^Δhep^). The deletion leads to a frameshift and early translation termination, resulting in a 57 amino acid truncated protein. Mrs2^fl/fl^ and Mrs2^Δhep^ mice were maintained in the Penn State College of Medicine animal facility following approval from the Institutional animal care and use committee. All mice were grouped according to sex, age, and genotype and used as required. Both male and female mice were used to isolate primary hepatocytes and mitochondria. For liver IR injury, male mice were used.

### Acute liver ischemia and reperfusion injury: hanging weight system:

We used portal triad occlusion and hanging weight system to induce acute liver ischemia-reperfusion injury as described previously^122^. The peritoneal cavity was exposed after a midline laparotomy and incision of the linea alba. The liver was kept wet and warm during surgery with a wet swab soaked with saline at 37°C. The stomach and duodenum were caudally displaced using a wet cotton tip swab to expose the portal triad and caudate lobe. The caudate lobe was gently separated from the left lobe, and the right lobe was then slightly shifted to clearly view the portal triad above the bifurcation of the right, median, and left lobes. Once visually identified, the needle, followed by a suture (7/0 nylon suture; Ethicon, Norderstedt, Germany), was placed under the portal triad, including the hepatic artery, hepatic vein, and common bile duct. The left end of the suture was then placed over the right pole, whereas the right end was placed over the left pole, and a weight of 1.5 grams was attached to each end. The surgical wound was closed using continuous muscle walls and skin sutures. After surgery, mice were allowed to recover for 1 h of reperfusion under a heating lamp. Sham-operated mice served as the control and underwent anesthesia, laparotomy, and exposure to the portal triad without I/R. All animals survived the surgical procedure, and no complications were observed with portal triad occlusion using the hanging-weight system or in control mice.

### Plasmids:

Human Mrs2 full length (Mrs2^WT^) and its mutants (Mrs2p^ΔGMN^ and Mrs2p^ΔMBS^) were custom synthesized as gBlock gene fragments from IDT Inc. and cloned into pCMV6-Entry Cloning Vector (Origene) for expression in mammalian system. The plasmids/clones were confirmed by restriction enzyme analysis (REA) and sequencing before use.

### Primary hepatocytes and cell lines:

Primary adult mouse hepatocytes were isolated from 10–12 week-old male and female animals using the two-step collagenase perfusion technique with slight modifications^123^. In brief, the liver was sequentially perfused with 50 ml of perfusion medium-I (DPBS containing 10mM HEPES, 0.05% w/v KCl, 5mM Glucose, 200 μM EDTA, pH 7.4) and 20 ml of perfusion medium-II (DPBS containing 30mM HEPES, 0.05% w/v KCL, 5mM Glucose, 1mM CaCl2, pH 7.4) containing liberase (250 μg). After perfusion, liver lobes were dissected, dissociated, and crude hepatocyte preparation was passed through a 70 μm cell strainer. The crude hepatocyte preparation was centrifuged at 50xg for 2 mins. The hepatocyte pellet was resuspended in 10 ml of attachment media twice, and viable hepatocytes were separated from dead cells by the Percoll gradient (90%) by centrifugation at 200xg for 20 mins (centrifuged with low acceleration and low brake to minimize trauma to hepatocytes). Following gradient purification, the hepatocytes were plated in culture dishes using the hepatocyte attachment medium (Williams E medium containing 1% (v/v) antibioticantimycotic solution (GIBCO), 1% (v/v) 200mM L-glutamine, 1% (v/v) non-essential amino acids, and 10% fetal bovine serum). After attachment, media was replaced with the hepatocyte culture medium (Williams E medium containing 1% (v/v) antibiotic-antimycotic solution (GIBCO), 1% (v/v) 200mM L-glutamine, and 1% (v/v) nonessential amino acids).

HEK 293 (ATCC# CRL 1573) cells were grown in Dulbecco’s modified Eagle’s medium (DMEM)/10% FBS, supplemented with 100 U/ml penicillin, 100 μg/ml streptomycin at 5% CO_2_ and 37⁰C. MISSION lentiviral particles (NM_020662.11348s1c1) carrying the shRNA (target sequence: CTTTGTCAGTAT AGGGAATTA) targeting 3’UTR were used to knock down Mrs2 in HEK293 cells. HEK293 cells (5×10^5^ per well) grown in 6-well plates were transduced with a lentivirus. Seventy-two hours post-transduction, cells were selected with puromycin (2 μg/ml) for 6–10 days until clonal. The puromycin-resistant clones were expanded and stored. The level of Mrs2 Knockdown was assessed by qPCR and Western blot analysis.

HEK293 (5×10^5^ per well) cells grown in 6-well plates were transfected with plasmids expressing Mrs2p^WT^, Mrs2p^ΔGMN^, and Mrs2p^ΔMBS^. Forty-eight hours post-transfection cells were selected with G418 sulfate (2 μg/ml) for 6–10 days until clonal. The G418-resistant clones were expanded and stored. The stable expression of Mrs2 was assessed by Western blot analysis.

### Western blotting:

Cell extracts from tissues/control and KO hepatocytes/HEK293 NegShRNA and Mrs2 KD cells were prepared using RIPA buffer (50 mM Tris-HCl, pH 7.4, 150 mM NaCl, 0.25% deoxycholic acid, 1 mM EDTA, 1% NP-40, protease inhibitor cocktail (Complete, Roche), and Halt phosphatase inhibitor cocktail (Thermo Scientific). Equal amounts of protein (25 μg/lane) were separated on 4–12% Bis-Tris polyacrylamide gel, transferred to a PVDF membrane using iBlot 2 PVDF regular stacks (Thermo Scientific) and probed with antibodies specific for Mrs2 (1:500, Novus biologicals: NBP2–34200), MCU (1:500, Cell signaling technology: 14997), MICU1 (1:500, Cell signaling technology: 12524), cytochrome c (1:5000, Santa Cruz: sc-13156), actin (1:5000, Santa Cruz: sc-47778), Total OXPHOS Rodent Cocktail (1:5000, Abcam: ab110413), MCUR1 (1:500, Cell signaling technology: 13706), HA-Tag-HRP Conjugate (1:2000, Cell signaling technology: 2999), Monoclonal ANTI-FLAG^®^ M2 (1:3000, Millipore Sigma: F1804), Phospho-PDH (1:500, Cell signaling technology: 31866), Pyruvate Dehydrogenase (1:500, Cell signaling technology: 2784) Phospho-AMPKα (Thr172) (1:500, Cell signaling technology: 2535), AMPKα (1:500, Cell signaling technology: 2532).

### Localization and orientation of Mrs2:

To test the localization of Mrs2 to the mitochondrial membrane, permeabilized HEK293 cells stably expressing Mrs2^WT^-Flag were permeabilized with digitonin (40 μg/ml). The permeabilized cells were exposed to mastoparan (20 μg/ml; a wasp venom peptide toxin that induces OMM permeabilization) or alamethicin (20 μg/ml; a fungal peptide that induces large pores in both mitochondrial membranes)^12^ for 5 mins. After permeabilization and treatment, cytosolic (supernatant) and mitochondrial (pellet) fractions were collected and subjected to Western blotting with antibodies specific for CytC, Mrs2, MCU, SPG7 (1:500, Novus biologicals: NBP2–01860), and CypD (1:1000, Santa Cruz: sc-376061). To test the orientation of Mrs2, mitochondria isolated from HEK293 cells stably expressing Mrs2^WT^-Flag were suspended in a hypotonic solution (5 mM sucrose, 5 mM HEPES, and 1mM EGTA (pH 7.2)) to swell and rupture the OMM. The suspension was centrifuged at 1000xg, and pelleted mitoplasts were resuspended in mitochondrial resuspension buffer (750 mM KCl, 100 mM HEPES, and 1 mM EGTA (pH 7.2)). The mitoplasts were treated with proteinase K digestion followed by Western blot analysis with antibodies specific for Mrs2, MCU, CypD, Tom20 (1:1000, Santa Cruz: sc-17764), and actin.

### Co-immunoprecipitation and Western Blot Analysis:

Cell extracts were prepared from transiently transfected HEK293 cells using RIPA buffer (50 mM Tris-HCl (pH 7.4), 150 mM NaCl, 0.25% deoxycholic acid, 1 mM EDTA, 1% NP-40, protease inhibitor cocktail (Complete: Roche and 1 mM PMSF). To study the interaction of MCU with MCU complex components in the presence of varying concentrations of Mg^2+^, HA-tagged MCU was co-transfected with Flag-tagged MCUR1, EMRE, MICU1, and MCUb. Following immunoprecipitation with HA antibody in the presence of varying concentrations of MgCl_2_, total cell lysates and immunoprecipitated materials were subjected to Western blot analysis. 10% of cell lysates were probed with Flag and HA antibodies to serve as inputs, and similarly, immunoprecipitated samples were probed with their corresponding antibodies to assess protein binding. To study the interaction of Mrs2 with MCU complex components, HAtagged Mrs2 was co-transfected with Flag-tagged MCU, MCUR1, EMRE, and MICU1. Co-immunoprecipitation and western blotting were performed as described above. To study whether the interaction of MCU and MICU1 still exists in the absence of matrix Mg^2+^, we co-transfected MCU-Flag and MICU1-HA in HEK293 NegShRNA and Mrs2KD cells.

### qPCR analysis:

Various mouse tissues were collected from WT/Mrs2^fl/fl^/Mrs2^Δhep^ mice and preserved in RNA later. mRNA was isolated from tissues using the PureLink RNA Mini Kit (Invitrogen, ThermoFisher Scientific). cDNA was prepared using the iScript^™^ cDNA Synthesis Kit (BIO-RAD, USA) following the manufacturer’s instructions. qPCR was performed using the PrimeTime^™^ Gene Expression Master Mix and PrimeTime predesigned probe for mouse Mrs2. Actin was used to normalize the mRNA levels. Mrs2 expression in HEK293 NegShRNA and Mrs2KD was analyzed by qPCR using the PrimeTime predesigned probe for human Mrs2. HPRT was used to normalize the mRNA levels.

### Simultaneous measurement of mMg^2+^/mCa^2+^ uptake and mitochondrial membrane potential in permeabilized cells:

An equal number of Mrs2^fl/fl^/Mrs2^Δhep^ /HEK293 NegShRNA/Mrs2KD cells (6 × 10^6^ cells) were washed in Ca^2+^/Mg^2+^ free PBS, pH 7.4, resuspended and permeabilized with 40 μg/ml digitonin in 1.5 ml of intracellular medium (ICM) composed of 120 mM KCl, 10 mM NaCl, 1 mM KH2PO4, 20 mM Hepes-Tris, pH 7.2 and 2 μM thapsigargin to block the SERCA pump. All the measurements were performed in the presence of 5 mM succinate. Δψm and extramitochondrial Ca^2+^ ([Ca^2+^]out) clearance as an indicator of mCa^2+^ uptake was achieved by loading the permeabilized cells with JC-1 (800 nM) and Fura2-FF (0.5 μM), respectively. Fluorescence was monitored in a multi-wavelength excitation dual-wavelength emission fluorimeter (Delta RAM, PTI). [Ca^2+^]out is represented as the excitation ratio (340 nm/380 nm) of Fura2-FF/FA fluorescence, and Δψm as the ratio of the fluorescence of J-aggregate (570 nm excitation/595 nm emission) and monomer (490 nm excitation/535 nm emission) forms of JC-1. A single or series of Ca^2+^ bolus (20, 10, or 1 μM) and mitochondrial uncoupler, CCCP (2 μM), were added at the indicated time points. All the experiments were performed at 37°C with constant stirring^27, 38, 124–128^.

To measure mMg2+ uptake, we performed a similar experiment using the ratiometric dye, Mag-Fura-2 tetra potassium salt. Mag-Fura-2 was calibrated, and the [mMg2+] was determined by [mMg2+]=Kd((R−Rmin)/(Rmax−R)) Where the dissociation constant Kd is 1.98 mM.

### Mitoplast patch-clamp recording:

#### I_MCU_ measurements:

Mitoplast patch-clamp recordings for MCU (I_MCU_) were performed at 30°C, as reported earlier^38, 125, 128^. In brief, freshly prepared mitoplasts from HeLa, HEK293 cells, or hepatocytes of Mrs2^fl/fl^ and Mrs2^Δhep^ mice were plated on the Cell-Tak–coated coverslips and mounted on the microscope. The mitoplasts were bathed in a solution containing 150 mM sodium gluconate, 5.4 mM KCl, 5 mM CaCl_2_, and 10 mM HEPES (pH 7.2). The pipette solution contained 150 mM sodium gluconate, 5 mM NaCl, 135 mM sucrose, 10 mM HEPES, and 1.5 mM EGTA (pH 7.2). After the formation of GΩ seals (20 to 35 MΩ), the mitoplasts were ruptured with a 200 to 400 mV pulse for 2 to 6 ms. After capacitance (2.5 to 3.0 pF) compensation, mitoplasts were held at 0 mV, and I_MCU_ was recorded with a voltage ramp (from −160 to 80 mV, 120 mV/s). The external/bath solution (5 mM Ca^2+^) was chosen based on our previous measurements^38, 125, 128^. Samples were discarded if the break-in took longer than 5 s after adding 5 mM Ca^2+^. I_MCU_ currents were recorded using an Axon200B patch-clamp amplifier with a Digidata 1320A acquisition board (pCLAMP 10.0 software; Axon Instruments). For measuring the effect of matrix Mg^2+^ on I_MCU_, varying concentrations of Mg^2+^ were present in the pipette solution.

#### I_Mrs2_ measurements:

Mitoplasts were prepared from Mrs2^fl/fl^ and Mrs2^Δhep^, as mentioned above. The mitoplasts were bathed in a solution containing Na-gluconate (150 mM), HEPES (10 mM) (pH 7.4 with N’-Methyl-Dglucamine (NMDG)), NaCl (5 mM), sucrose (135 mM), and 5 mM MgCl_2_. In some measurements, 200 nM ruthenium red (Ru360) or 1 μM hexamine cobalt(III)chloride was added to the bath. I_Mrs2_ was recorded as described above.

### Protein purification and planar lipid bilayer recordings for single-channel activity measurements:

The construct for Mrs2 was expressed in HEK 293 cells and purified using the EZ view Red ANTI-FLAG M2 Affinity Gel (Sigma), according to the manufacturer’s protocol. A silver-stained gel assessed the purity of isolated Mrs2 protein. Planar lipid bilayer recordings were performed in 150 mM NMDG and 10 mM HEPES solution buffered at (pH 7.4); supplemented with 20–104 mM Mg^2+^ during recordings on the cis side. α-L-phosphatidylcholine (Sigma) was used for forming the bilayer membrane. ePatch amplifier (Elements) was used for lipid bilayer recordings. Purified Mrs2 (5 μg, final concentration) was used for single-channel recordings, and Co^2+^ (1–5 mM, final concentration) was added into the bath during the recordings without perfusion. pCLAMP-10 software was used for electrophysiology data acquisition and analysis (Molecular devices). All current measurements were adjusted for the holding voltage assuming a linear current-voltage relationship: The resulting conductances are expressed in pS according to the equation G = I/V where G is conductance in pS, V is the membrane holding voltage in mV, and I is the peak membrane current in pA after subtraction of the baseline electrode leak current. Group data were quantified in terms of conductance. All population data were expressed as mean ± SEM.

### ATP Measurement:

Total ATP abundance was assessed in Mrs2^fl/fl^/Mrs2^Δhep^ /HEK293 NegShRNA/Mrs2KD cells using CellTiter-Glo^®^ luminescent assay as per manufacturer’s instructions^27, 38, 125–128^.

### Mitochondrial Oxygen Consumption Rate:

Intact Mrs2^fl/fl^/Mrs2^Δhep^ /HEK293 NegShRNA/Mrs2KD cells were subjected to oxygen consumption rate (OCR) measurement at 37°C in an XFe24 extracellular flux analyzer (Seahorse Bioscience). Cells (2.5 × 10^5^) were sequentially exposed to 2 μM oligomycin, 0.5 μM FCCP, and 0.5 μM rotenone plus antimycin A at indicated time points to measure basal and maximal respiration, ATP production, proton leak, spare respiratory capacity, and non-mitochondrial respiration as described previously^27, 38, 125–128^.

### Microscopy, live cell imaging, and confocal measurements:

(i) Mitochondrial localization of Mrs2, confocal imaging: 0.5×10^6^ HeLa cells grown on 0.2% gelatin-coated glass coverslips were co-transfected with Cox8-mRFP and Mrs2-GFP. Forty-eight hours post-transfection, confocal images (Leica SP8) were obtained at 561 and 488 nm excitation using a ×63 oil objective. Overlap of RFP and GFP signals was quantified using Leica LAS-X software. (ii) Mitochondrial localization of Mrs2: SIM super resolution microscopy. 0.5×10^6^ HeLa cells grown on 0.2% gelatin-coated glass coverslips were co-transfected with Cox8-mRFP and Mrs2-GFP. Forty-eight hours post-transfection, cells were fixed with 4% paraformaldehyde and stored at 4°C until imaging. The slides were imaged under a Nikon N-SIM/STORM SR microscope in SIM mode with an SR Apo TIRF100x, NA1.49 objective, and the images were captured with an Andor DU 897x EMCCD camera. The GFP channel was excited by a 488 nm laser to collect an emission peak at 522 nm, while the RFP was excited by a 561 nm laser to obtain an emission peak at 605 nm. Images taken in 3D SIM mode were processed and reconstructed with Nikon Elements. The reconstructed images were analyzed for colocalization in Nikon Elements and ImageJ. (iii) TMRM and Calcein fluorescence (Time-series/intact cell) measurements: 0.5×10^6^ hepatocytes isolated from Mrs2^fl/fl^ and Mrs2^Δhep^ mice grown on 0.1% collagen-coated glass coverslips were stained with the Δψm indicator TMRM or PTP opening indicator calceinAM. Coverslips were mounted in an open perfusion micro incubator (PDMI-2; Harvard Apparatus) and imaged at 561 or 488 excitations using a ×63 oil objective. After baseline measurements, ionomycin (5 μM) was added, and changes in TMRM or calcein fluorescence were recorded every 3 s (Leica SP8) for 10 mins. The loading and imaging buffer contained cobalt chloride (CoCl_2_; 1mM) for calcein measurement. The presence of CoCl_2_ quenched the calcein fluorescence in the cytosol and nucleus, leaving the mitochondrial fluorescence intact. As the PTP opens, CoCl_2_ enters the mitochondrial matrix and quenches calcein fluorescence. The decrease in calcein fluorescence after ionomycin treatment was measured using the Leica LAS-X software. (iv) Confocal imaging of mitochondrial Δψm: 0.5×10^6^ hepatocytes isolated from Mrs2^fl/fl^ and Mrs2^Δhep^ mice grown on 0.1% collagen-coated glass coverslips were stained with the Δψm indicator TMRM and dihydrorhodamine 123 (DHR123). Coverslips were mounted in an open perfusion micro incubator (PDMI-2; Harvard Apparatus) and imaged at 561 or 488 excitations using a ×63 oil objective. Images were analyzed, and the mean TMRM fluorescence was quantified using Image J software (NIH). (v) Measurement of cytosolic Mg^2+^ dynamics. 0.5×10^6^ hepatocytes isolated from Mrs2^fl/fl^ and Mrs2^Δhep^ mice grown on 0.1% collagen-coated glass coverslips were stained with MagGree-AM and MitoTracker Red. Coverslips were mounted in an open perfusion micro incubator (PDMI-2; Harvard Apparatus) and imaged at 488 and 561 excitations using a ×63 oil objective. After baseline measurements, glucagon (10 μM) and 2 mM Mg^2+^ were added at the indicated time. MagGreen fluorescence changes (cytosolic and mitochondrial) were recorded every 3 s (Leica SP8) for 10 mins. The rate of cytosolic MagGreen decay and the mitochondrial peak fluorescence was measured using Leica LAS-X software.

### Mitochondrial isolation and swelling assay:

Hepatocytes isolated from Mrs2^fl/fl^ and Mrs2^Δhep^ were homogenized in ice-cold mitochondrial isolation buffer (10 mM sucrose, 200 mM mannitol, 5 mM HEPES, and 1 mM EGTA, pH 7.4) containing 1 mg/ml fatty acid-free bovine serum albumin. The homogenate was centrifuged for 10 min at 1000 x g, and the supernatant was centrifuged again at 14,000 x g for 10 min. The mitochondrial pellets were washed twice and centrifuged at 11,200 xg. The isolated mitochondria (1 mg protein) were added to 0.2 ml of mitochondrial swelling buffer (70 mM sucrose, 230 mM mannitol, 3 mM HEPES, 2 mM Trisphosphate, 5 mM succinate). Mitochondrial swelling was measured by the decrease in absorbance at 540 nm after adding Ca^2+^ (250 μM)^27^.

### Flow Cytometry and PI Staining:

Hepatocytes isolated from Mrs2^fl/fl^ and Mrs2^Δhep^ were treated with ionomycin (25 μM) for 6 hr. For necrotic cell death measurement, cells were stained with propidium iodide. The cells were analyzed with the BD LSR Fortessa (BD Biosciences). Relative PI staining was plotted on a logarithmic scale using Flow-Jo software^27, 38^.

### Size Exclusion Chromatographic Analysis of MCU Complex:

Gel filtration was performed by fast protein liquid chromatography (ÄKTA Pure FPLC; GE Healthcare), and the Superdex 200 10/300 column was equilibrated with PBS. Column calibration was carried out with a gel filtration protein standards kit (Bio-Rad). Cleared lysates from HEK293 NegShRNA and Mrs2KD cells stably expressing MCU-FLAG were directly loaded onto a Superdex 200 FPLC column at a 0.5 ml/min flow rate. Forty fractions were collected, concentrated, and fractionation of MCU complex was assayed by Western blot analysis^27, 125^.

### Plasma enzymatic and cytokine measurements:

Plasma aspartate (AST) and alanine (ALT) aminotransferase activities were measured using a commercially available kit (Cayman) as per the manufacturer’s instruction. We used Mouse Cytokine Array Panel A (Proteome ProfilerTM Array; R&D systems) to determine the relative levels of 40 mouse cytokines.

### Histological assessment of damage:

The median and left liver lobes were harvested and fixed in 4% formalin. Fixed tissues were subsequently sectioned, ten μm thick, collected on (+) charge slides, and stained with hematoxylin and eosin. Examination and scoring of each lobe were carried out by a pathologist who was blinded to the experimental group.

### Quantification and statistical analysis:

Data from multiple experiments (≥3) were quantified and expressed as Mean ± SE, and differences between groups were analyzed using the two-tailed paired Student’s t-test or, when not normally distributed, a nonparametric Mann–Whitney U-test (Wilcoxon Rank-Sum Test) for two groups. The data were computed with GraphPad Prism version 9.0 or SigmaPlot 11.0 software. Differences in means among multiple datasets were analyzed using one-way ANOVA with Tukey correction performed. A P ≤ 0.05 was considered significant in all analyses.
